# Methicillin-Sensitive Staphylococcus aureus Bacteremia, Septic Arthritis, and Pyomyositis in a Young Male: A Case Report and Review of the Literature

**DOI:** 10.7759/cureus.77022

**Published:** 2025-01-06

**Authors:** Hana Shah, Aran Thiravialingam, Aditi Kumar, Lexie Mesa-Morales, Lorena Bonilla

**Affiliations:** 1 Department of Translational Medicine, Florida International University, Herbert Wertheim College of Medicine, Miami, USA; 2 Baptist Health Medical Group, Baptist Health South Florida, Miami, USA

**Keywords:** fatty liver disease, methicillin-sensitive bacteremia, pyomyositis, sacroiliac joint septic arthritis, staphylococcus aureus

## Abstract

We share a case of a 38-year-old male with a history of hypertension and metabolic dysfunction-associated steatotic liver disease (MASLD) who was admitted for septic arthritis of the left sacroiliac joint, pyomyositis, and associated methicillin-sensitive *Staphylococcus aureus* (MSSA) bacteremia. The patient presented with left hip pain, fever, tachycardia, and leukocytosis. A physical exam revealed left lateral hip tenderness and limited range of motion. Lumbar spine magnetic resonance imaging (MRI) revealed left sacroiliac septic arthritis, inflammation of multiple muscles consistent with pyomyositis, and a presacral abscess. Blood cultures and ​polymerase chain reaction results confirmed MSSA bacteremia, though no common predisposing risk factors were identified. The abscess was aspirated and the patient was treated with oxacillin and cefazolin. He showed clinical improvement with stable leukocytosis and was discharged on cefazolin via a peripherally inserted central catheter. Follow-up included a referral to rheumatology and a repeat of lumbar spine MRI. This case underscores the challenges in diagnosing MSSA bacteremia, especially in the absence of typical risk factors, and emphasizes the critical role of clinical suspicion and appropriate treatment strategies.

## Introduction

Pyomyositis is a skeletal muscle infection that is typically caused by *Staphylococcus aureus* [1,2]. With the potential to result in abscess formation, *S. aureus* bacteremia is associated with significant morbidity and mortality. Complications of pyomyositis include septic arthritis or osteomyelitis from the contiguous spread of infection to an adjacent joint or bone [3]. Patients commonly complain of joint and bone pain; however, these symptoms mimic that of other conditions like muscle contusion and necrotizing fasciitis. The rarity of pyomyositis combined with the non-specificity of its symptoms often leads to delays in treatment. As such, a high clinical suspicion is necessary for timely diagnosis. Here, we explore the clinical case of a patient who presented with pyomyositis involving multiple muscles, a large presacral abscess, and septic arthritis of the left sacroiliac joint, with methicillin-sensitive *Staphylococcus aureus* (MSSA) bacteremia as the causative agent.

## Case presentation

A 38-year-old male with a past medical history of hypertension and metabolic dysfunction-associated steatotic liver disease (MASLD) presented to the hospital with a two-day history of left hip pain. He had a fever and chills that started the day prior to admission. He denied trauma to the area, loss of motor or sensory function, urinary or bowel incontinence, and inflammatory skin conditions. The review of systems was unremarkable. There was no history of tobacco, alcohol, or illicit drug use, and the patient had no sick contacts or recent travel. On admission, he was taking oral enalapril 20 mg daily.

The physical exam was notable for left lateral hip tenderness and limited range of motion secondary to pain. On arrival, the patient was febrile (38.3°C), tachycardic (140 bpm), and hypertensive (171/130 mmHg). Tables 1-4 summarize the laboratory studies performed, including liver function tests (LFTs), autoimmune studies, inflammatory markers, and urinalysis. The results were significant for WBC (15.28 K/uL) and hyperglycemia (161 mg/dL). Polymerase chain reaction (PCR) analysis of the blood culture confirmed the presence of *Staphylococcus aureus*. The time to positivity (TTP) for the initial blood cultures was 72 hours, which has been associated with prognostic implications in bacteremia [4]. Follow-up blood cultures obtained 48 hours later were sterile.

**Table 1:** Complete metabolic panel. As liver function tests were unremarkable on admission, the test was not repeated. SGOT: serum glutamic oxaloacetic transaminase; SGPT: serum glutamic pyruvic transaminase.

Lab finding	On admission (4/21/2024)	At discharge (5/1/2024)	Reference range
Sodium (mEq/L)	132	131	136-145
Potassium (mEq/L)	4.1	4.4	3.5-5.1
Chloride (mEq/L)	96	100	98-107
Carbon dioxide (mEq/L)	26	25	21-32
Glucose (mg/dL)	161	110	70-126
Blood urea nitrogen (mg/dL)	11	18	7-18
Creatine (mg/dL)	1.27	0.99	0.6-1.3
Anion gap (mEq/L)	10	6	2-15
Albumin (g/dL)	3.8	-	3.4-5
Total protein (g/dL)	7.9	-	6.4-8.2
Calcium (mg/dL)	9	9.9	8.5-10.1
Total bilirubin (mg/dL)	0.9	-	0.2-1
Direct bilirubin (mg/dL)	0.2	-	0-0.2
Indirect bilirubin (mg/dL)	0.8	-	0.3-1.9
Alkaline phosphatase (U/L)	40	-	50-136
Aspartate aminotransferase (AST, SGOT)	19	-	8-37
Alanine aminotransferase (ALT, SGPT)	39	-	16-65
Lactic acid (mmol/L)	2.4	-	0.5-2.2

**Table 2:** Complete blood count.

Whole blood	On admission (4/21/2024)	At discharge (5/1/2024)	Reference range
WBC (K/ uL)	15.28	14.38	3.4-11
RBC (uL)	5.01	4.82	4-5.7
Hemoglobin (g/dL)	14.5	14.0	13-17.2
Hematocrit (%)	42.9	41.2	38-50
Platelet (K/ uL)	164	499	130-360

**Table 3:** Autoimmune studies and inflammatory markers.

Laboratory test	On admission (4/21/2024)	At discharge (5/1/2024)	Reference range
A1c (%)	5.6	-	4.8-5.6
Creatine kinase (U/L)	223	-	26-308
HIV	Negative	-	Non-reactive
Rheumatoid factor	Negative	-	Negative
Sedimentation rate (mm/hr)	10	-	0-20
C-reactive protein (mg/L)	202.0	32.5	0-10

**Table 4:** Routine urinalysis. Routine urinalysis was completed on admission; however, the patient did not show signs and symptoms of a urinary tract infection, thus it was not repeated at discharge.

Urinalysis	On admission (4/21/2024)	At discharge (5/1/2024)	Reference range
Glucose (mmol/L)	100	-	0-0.8
pH	6.5	-	5-8
Protein (mg/dL)	30	-	0-14
Specific gravity	1.02	-	1.005-1.030
Nitrite	Negative	-	Negative
Leukocyte esterase	Negative	-	Negative
WBC (WBC/hpf)	2-5	-	2-5
RBC (RBC/hpf)	5-10	-	4

**Figure 1:**
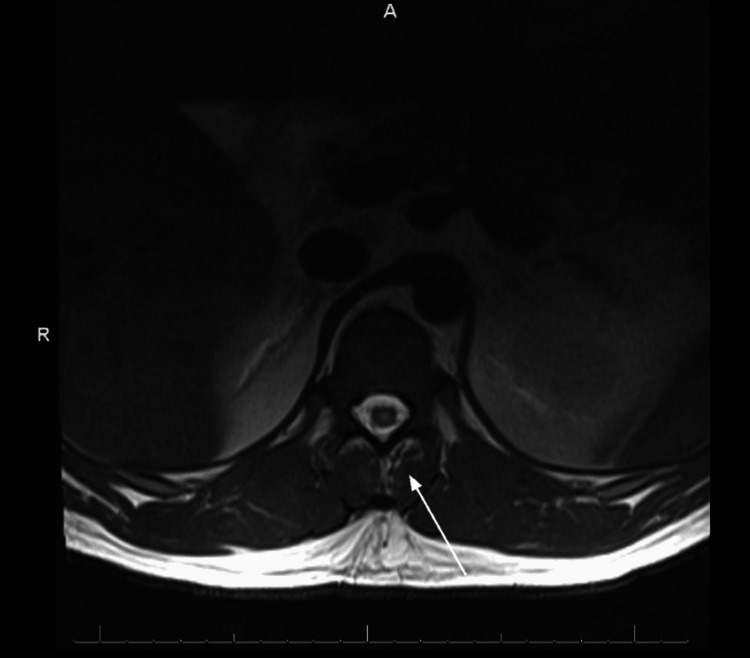
Lumbar spine MRI showing enhancement of the posterior paraspinal muscles on the left side at the L4-L5 level. No osseous enhancement or abscess was present.

**Figure 2:**
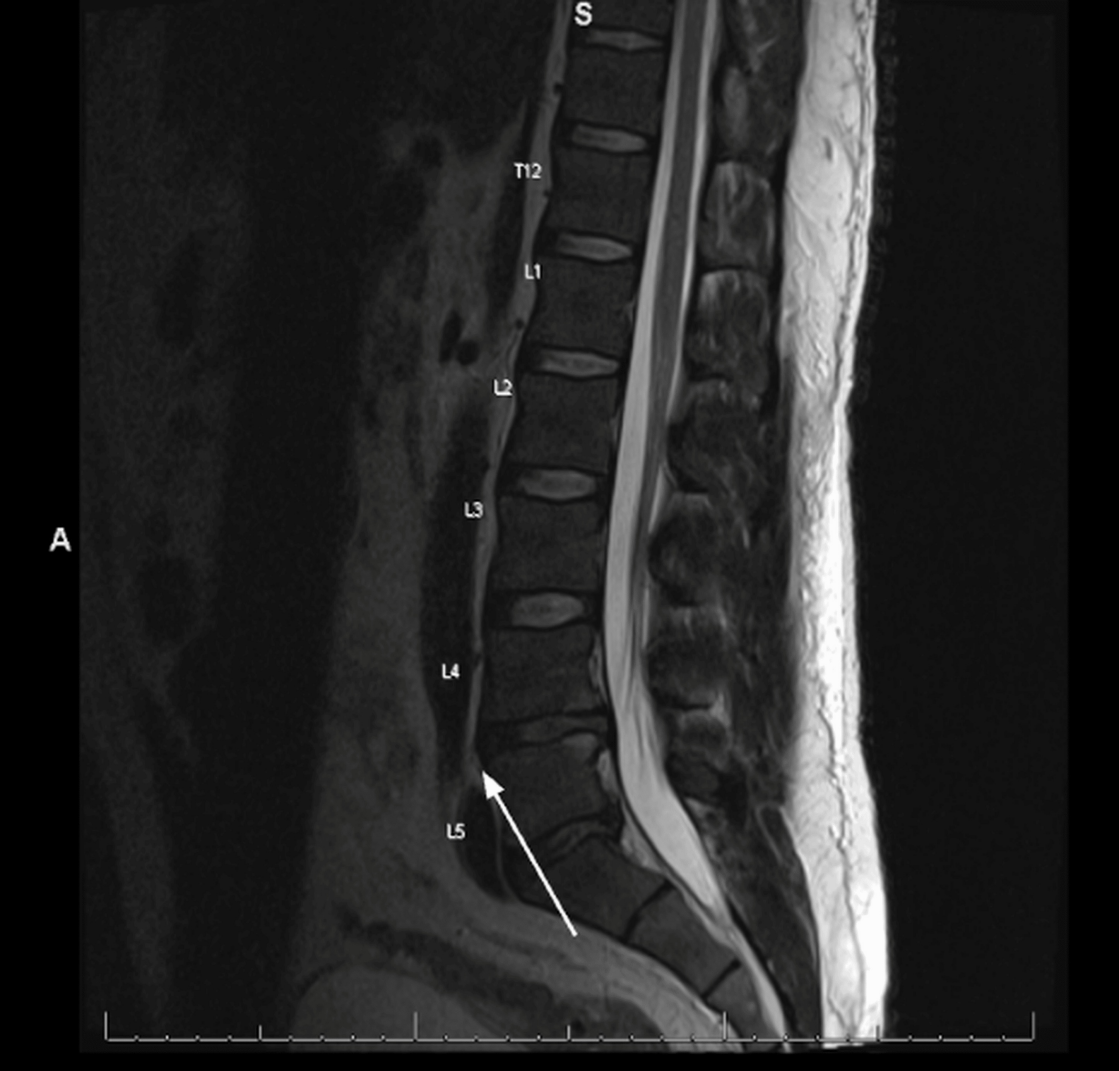
Lumbar spine MRI showing questionable associated epidural enhancement at the L5-S1 level, likely infectious in nature. No abscess or drainable collection was noted.

**Figure 3:**
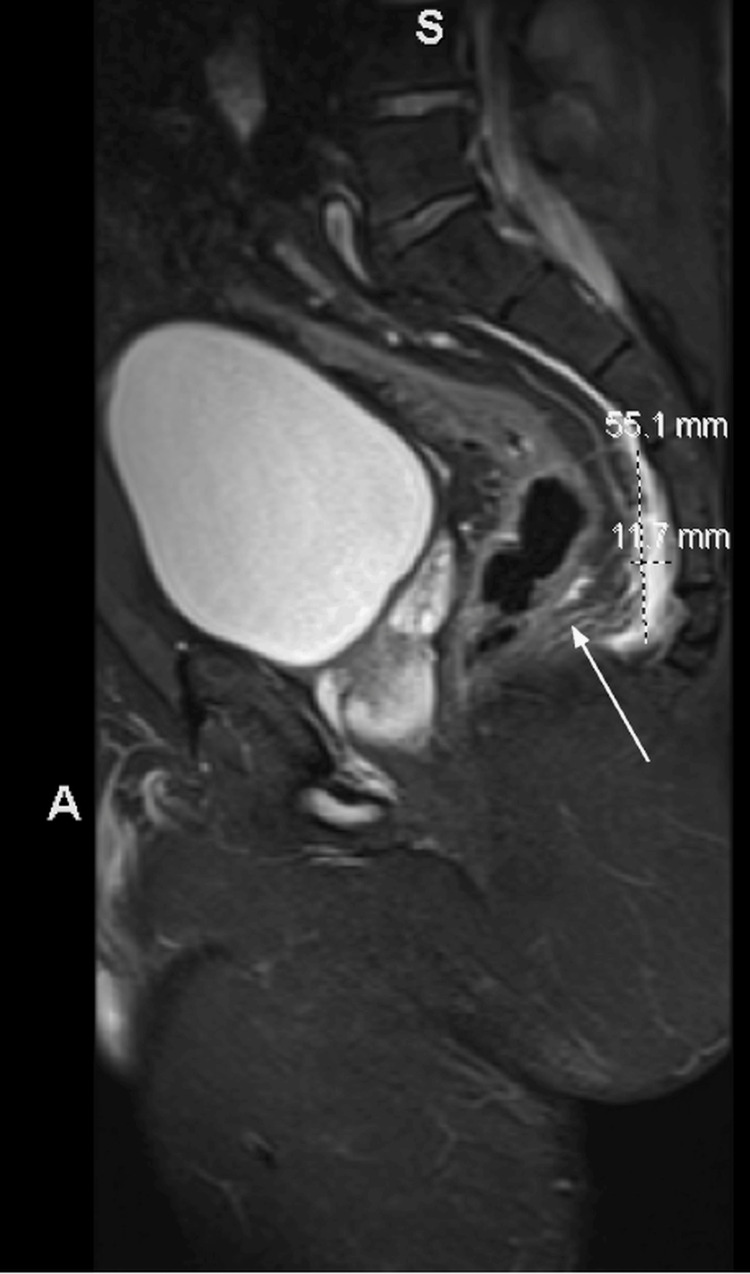
MRI of the pelvis with and without contrast showing the 5.5 x 1.2 x 5.7 cm presacral abscess formation. Of note, there was an intradural extension at the level of the left S1 and S2 neural foramen. Phlegmonous change over the lateral aspect of the left gluteus maximus muscle belly deep to the iliotibial band with small-volume abscess formation. No evidence of acute osteomyelitis.

**Figure 4:**
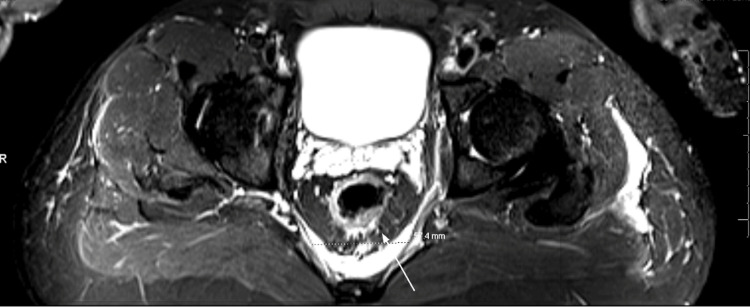
Irregular edema and enhancement in the left gluteus maximus, piriformis, iliacus, posterior psoas, and left paraspinal muscles, indicative of pyomyositis.

Chest radiograph, bilateral lower extremity Doppler ultrasound, and transthoracic echocardiogram were unremarkable. A lumbar spine magnetic resonance imaging (MRI) with and without contrast exhibited enhancement of the posterior left paraspinal muscles with degenerative changes and foraminal narrowing at L4-L5 (Figures 1, 2). Subsequently, an MRI of the pelvis with and without contrast suggested findings of a left sacroiliac joint septic arthritis, with 5.5 x 1.2 x 5.7 cm presacral abscess formation (Figure 3). Irregular edema and enhancement throughout the left gluteus maximus, piriformis, iliacus, posterior psoas, and left paraspinal musculature were seen - highly suggestive of pyomyositis (Figure 4). There was an extension of the infection at the level of the left S1 and S2 neural foramen, along with inflammation and a small abscess in the left gluteus maximus muscle. No signs of acute osteomyelitis were present.

Thus, the patient was diagnosed with MSSA bacteremia and left sacroiliac joint septic arthritis. On the fifth day, the presacral abscess was aspirated by the interventional radiology team, resulting in 1 mL of clear yellow fluid that was sent for culture analysis. Results remained negative. The patient was evaluated by the infectious disease (ID) physician who recommended a treatment regimen of oxacillin 2 g intravenously every four hours. The patient's leukocytosis remained stable at 14 K/uL and he had no episodes of fever or clinical deterioration. To evaluate for endocarditis via transesophageal echocardiogram (TEE), the cardiology team was consulted; however, the patient declined the procedure. On the eighth day, the patient’s treatment regimen was modified to cefazolin 2 g intravenously every eight hours. Upon discharge, the patient was prescribed cefazolin 2 g intravenously every eight hours for six weeks via a peripherally inserted central catheter (PICC) line. A repeat lumbar spine MRI was scheduled at the end of treatment. A referral to rheumatology was in place for further management of septic arthritis in the sacroiliac joint.

## Discussion

MSSA bacteremia is a global healthcare concern that is associated with poor clinical outcomes and can cause a range of organ-specific infections [5]. This report provides a detailed account of a 38-year-old male who experienced MSSA bacteremia with complications, including sacroiliac joint septic arthritis, a presacral abscess, and pyomyositis, occurring in the absence of common predisposing factors. It sheds light on the various aspects of managing infectious diseases, including diagnostic difficulties and treatment approaches.

Septic arthritis of the sacroiliac joint is uncommon and makes up 1-2% of all septic arthritis cases [6]. Those with a history of intravenous drug use, infective endocarditis, hemoglobinopathy, immunosuppression, and cutaneous or respiratory infections are particularly at risk of infectious sacroiliitis [7]. The condition is characterized by symptoms like fever, lumbogluteal pain, and limited mobility. However, the clinical manifestations may lead to delayed diagnosis and treatment. Thus, it is recommended that an MRI of the spine is completed promptly after recognizing signs of an infectious process [6,7]. Based on the patient's medical history, the infection likely spread hematogenously, increasing the risk of central nervous system involvement or metastatic infection [8]. It is important to note that this patient did not have the usual risk factors, as mentioned above, associated with the condition. Prompt and accurate diagnosis is necessary to avoid long-term complications such as chronic pain, joint dysfunction, and significant morbidity [9].

*Staphylococcus aureus* is the main pathogen involved in the development of purulent infectious myositis (PIM), a pyogenic infection of skeletal muscles that is accompanied by abscess formation and is endemic to tropical regions [10]. Its pathogenesis involves the seeding of axial skeletal muscles in the setting of bacteremia. Many have suspected that this seeding occurs in the setting of a pre-existing muscular strain or trauma. PIM from a *S. aureus* spontaneous bacteremia account for less than 0.5% of cases [11]. The clinical presentation typically involves localized muscle pain, swelling, and systemic signs of infection, which can make early diagnosis quite challenging. MRI is commonly used to detect pyomyositis as it can precisely demarcate the extent of the disease, allowing for prompt management [10].

The patient’s MRI results revealed enhancement in the piriformis muscle, posterior psoas and left paraspinal muscles, iliac crest muscle, and left gluteal muscle, strongly suggesting pyomyositis. His clinical course was complicated by the development of a presacral abscess, measuring 5.5 x 1.2 x 5.7 cm. In general, presacral abscesses may be difficult to drain, given their proximity to structures such as pelvic bones, iliac vessels, bowel, and bladder [12]. Thus, interventional radiology was required to perform aspiration. The intervention provided immediate diagnostic clarity. Through successful aspiration and the administration of appropriate antibiotic therapy, there was an improvement, and no clinical worsening was noted.

​​It is important to consider the implications associated with declining a TEE to rule out endocarditis. *S. aureus* bacteremia is known to have a high association with endocarditis, particularly in patients with persistent bacteremia or embolic phenomena [13]. However, the lack of clinical signs of endocarditis and the patient's positive response to treatment helped alleviate this concern to a certain degree.

Effective management of MSSA bacteremia requires prompt identification and administration of appropriate antimicrobial therapy. While there are different treatment options, beta-lactam antibiotics, such as oxacillin and cefazolin, are the antibiotics of choice for MSSA bacteremia [14]. The ID team played an active role in this patient’s case. With the team adhering to evidence-based care practices, the patient completed early blood cultures with constant monitoring of WBC. Detailed recommendations for antibiotic regimes and further imaging were routinely provided. Thus, while adhering to guidelines, the patient’s treatment regimen involved intravenous oxacillin, followed by intravenous cefazolin for outpatient therapy. The patient's clinical improvement, stable leukocytosis, and sterile blood cultures suggest that the antibiotic regimen was effective. The expertise provided by ID consultations has been historically linked to improved outcomes and reduced mortality in *Staphylococcus aureus* bacteremia cases​ [15]. This case highlights the importance of thorough assessment while encouraging follow-up in patients with complicated bacteremia to prevent recurrence and ensure complete resolution of the infection.

## Conclusions

Given that this patient developed spontaneous septic arthritis with no underlying history of any trauma or inflammatory skin condition, physicians should understand the importance of rapidly identifying and treating MSSA bacteremia. Localized inflammation of the sacroiliac joint may have been secondary to hematogenous spread. Further studies are needed to investigate the presentation of MSSA bacteremia in individuals who lack common predisposing factors.
